# Case Report: Giant cholesterol granuloma in the anterior mediastinum

**DOI:** 10.3389/fcvm.2024.1359731

**Published:** 2024-04-26

**Authors:** Milica Ludoski, Igor Zivkovic, Petar Milacic, Novica Boricic, Slobodan Micovic, Milovan Bojic, Zoran Tabakovic

**Affiliations:** ^1^ Cardiac Surgery Department, Dedinje Cardiovascular Institute, Belgrade, Serbia; ^2^ Faculty of Medicine, University of Belgrade, Belgrade, Serbia; ^3^ Institute of Pathology, Faculty of Medicine, University of Belgrade, Belgrade, Serbia

**Keywords:** cholesterol granuloma, mediastinum, open heart surgery, CABG, tumor

## Abstract

Cholesterol granuloma is a rare entity, which can develop in many regions of the body, accounting at most 1% of all mediastinal tumors. Etiology of this granuloma is still not clearly understood. The gold standard choice of treatment for cholesterol granuloma is total surgical resection. Symptomatic mediastinum granuloma can be easily diagnosed, but if mass effect is not evident then diagnosis of this tumor is really challenging. We present a rare case of huge cholesterol granuloma in the anterior mediastinum of the patient who underwent on elective coronary artery graft bypass surgery.

## Background

Cholesterol granuloma is a rather uncommon entity. It is a benign tumor that can form in many different parts of the body, accounting for 1% of all mediastinal tumors (1). It is a reactive lesion caused by cholesterol crystals and foreign body giant cells (2). Because of its rarity, preoperative identification of mediastinal cholesterol granuloma might be difficult, however it is usually discovered incidentally during cardiac surgical operations (1). We describe an unusually large cholesterol granuloma in the patient's anterior mediastinum after elective heart surgery.

## Case description

**Figure 1:**
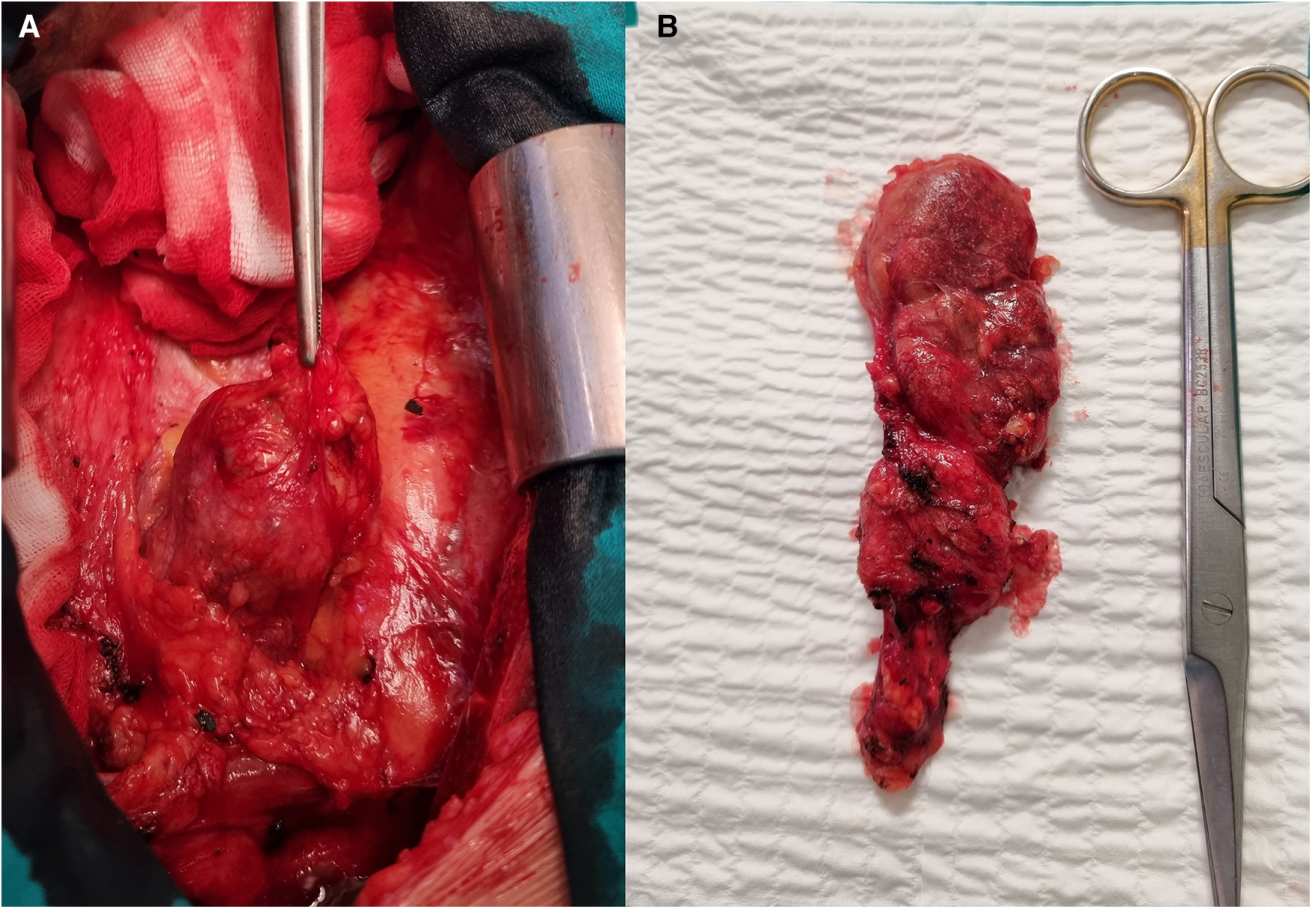
Surgical view on the tumorous mass. (**A**) Tumor into the mediastinum, view through the median sternotomy. (**B**) Completely extirpated tumor.

**Figure 2:**
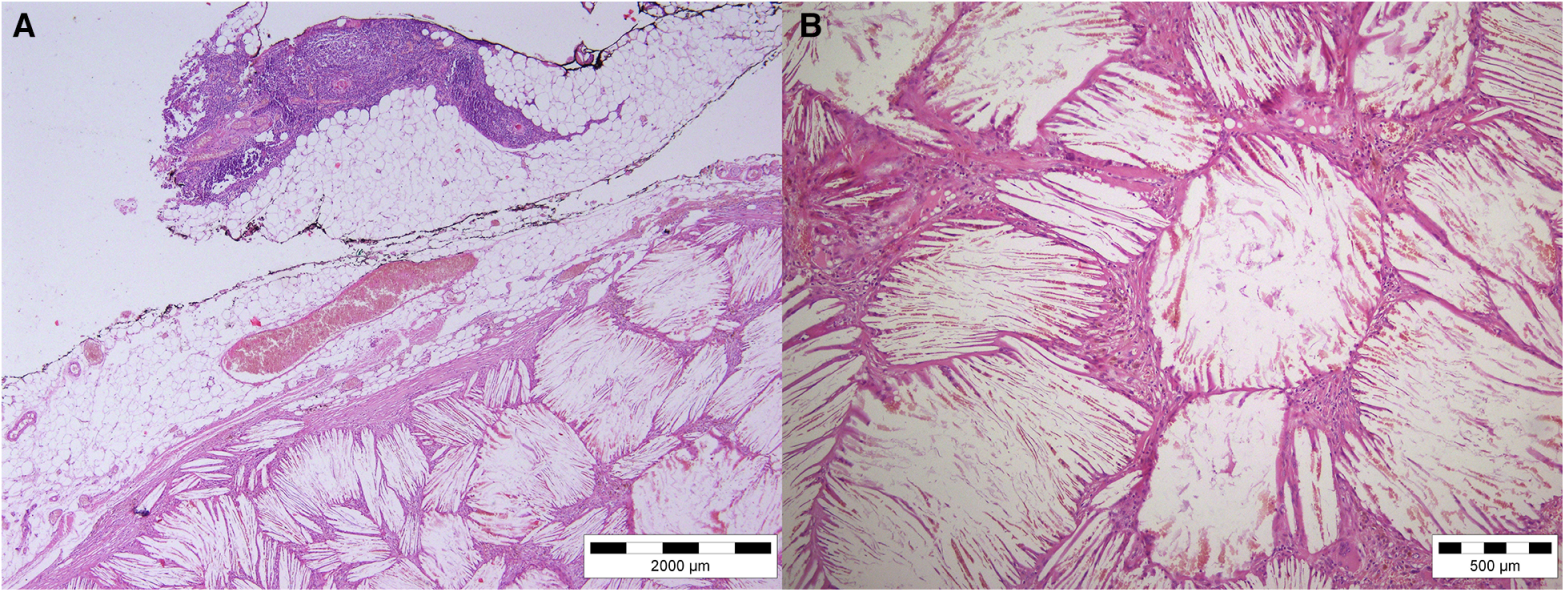
Hematoxylin—eosin staining of tumor tissue. (**A**) H&E magnification 4×. (**B**) H&E magnification 10×.

A 46-year-old man was referred to our institution for an coronary artery bypass graft surgery. He had angina symptoms such as shortness of breath and chest discomfort. Previously, there have been hypertension, dyslipidemia, and spontaneous pneumothorax. There was no prior history of chest trauma. The physical exam, laboratory results, and chest radiography were all unremarkable. We discovered a tumorous mass in the anterior mediastinum after a medial sternotomy. It was found in the mediastinal fat tissue in the projection of the thymus adherent on the pericardium. There were no complaints of a mass impact or structural deterioration in the mediastinum. A 11 × 4 cm multinodular tumor encapsulated in brown and yellow hue on the surface (Figures 1A,B). The tumor was completely surgically removed, followed by elective coronary artery bypass graft surgery. The patient's postoperative course was unremarkable. A histopathological investigation revealed a large number of cholesterol crystals enclosed by hyalinized collagen fibers. At increased magnification, macrophages, foreign-body type large cells, and dystrophic calcification were seen. There was no indication of cytologic atypia, mitotic activity, or necrosis, excluding the possibility of malignancy (Figures 2A,B).

## Discussion

Cholesterol granuloma in the anterior mediastinum in the thymus projection is an uncommon occurrence (1). It develops as a result of a foreign-body giant cell reactivity to cholesterol crystals. Cholesterol granuloma is histologically composed of granulation tissue with masses of cholesterol crystals surrounded by giant cells, macrophages, and lymphocytes. This aspect is significant since it is something that must be mentioned on a pathohistological test to identify cholesterol granuloma (2). The origin and pathophysiology of this granuloma are currently unknown. It might manifest as a result of chronic inflammation or as a result of trauma or a chest injury (2, 3). Chronic inflammation causes cell damage and hemolysis. Hemolysis liberates cholesterols from deteriorated cell membranes, allowing them to crystallize. Cholesterol crystals activate macrophages and foreign-body type giant cells, causing these cells to aggregate and cause persistent inflammation and granuloma development (4). Systemic variables such as dyslipidemia and hypertension are risk factors for the development of cholesterol granuloma because they can disrupt microcirculation, resulting in microhemorrhage and cell degeneration (1). Microhaemorrhage and cell degeneration also maintain chronic inflammation in the fat tissue what lead to granuloma formation. Granuloma formation is some kind of a tissue response. All of these systemic issues are affecting our patient. Cholesterol granuloma can form in any area of the body where there is cholesterol crystal deposition. However, granuloma is most commonly found at the petrous apex of the temporal bone and the middle ear, where it is associated with persistent inflammation. Aside from that, only a few instances have been described in the anterior mediastinum (2, 4). Cholesterol granuloma development in the anterior mediastinum has been observed as a multilocular thymic cyst against a background of chronic inflammation of the thymic parenchyma and in the case of thymic seminoma. However, just one example of this kind of granuloma development in the upper mediastinum has been recorded (3). Symptoms and clinical presentation vary and may be owing to space occupying effects on adjacent tissues, or the granuloma may be asymptomatic and only be detected accidentally. The diagnosis, management, and therapy of cholesterol granuloma are mostly guided by the location, size, and appearance of the tumor. Preoperative diagnosis of cholesterol granuloma in the mediastinum might be difficult and challenging. It can be found incidentally on imaging done for some different reasons and it is important to have this as a potential differential diagnosis. The following imaging techniques are used for diagnosis: computed tomography (CT), magnetic resonance imaging (MRI), positron emission tomography (PET), and chest radiography (posterior-anterior and lateral projection). CT scan shows well-circumscribed tumour mass. MRI often shows a low signal intensity on T1- and T2-weighted images due to numerous collagen fibers in granuloma. On positron emission tomography (PET) granuloma is positive. PET scan shows high fluoro-2-deoxy-D-glucose uptake in tumour like other malignant mediastinal tumours. It would be easier to evaluate and potentially diagnose this kind of tumour with preoperative imaging with CT or MRI, but in our case, our patient did not have any unusual symptoms in spite of angina symptoms. Because of that and because of patient's age, we did not perform any of this, neither CT, nor MRI. CT or MRI are not routinely performed in patients with coronary disease who underwent only coronary artery bypass grafting surgery. Hongo et al. describe a case of cholesterol granuloma in the antherior mediastinum. Their patient was man with an incidental finding of anterior mediastinal mass by PET scan. (5) Their patient was asymptomatic, without comorbidities, but he was rugby player with often chest trauma in history. In their case often patient's trauma was important and one of the main factors that lead to formation of this granuloma in mediastinum. Although there are a few diagnostic procedures available, due to its rarity and radiological appearances that resemble more frequent mediastinal tumors, an exact and correct diagnosis cannot be achieved without a pathohistological exam (1). Cholesterol granuloma is a space-occupying lesion that is benign, with no erosion of tissues in the mediastinum, and it can be discovered as an accidental discovery during cardiac surgery (4). The procedure of choice is total surgical resection. Weissferdt et al. investigated four individuals who had mediastinal cholesterol granuloma. Three years following full surgical excision of their lesions, they were followed up on. Clinical follow-up on the four patients has revealed no indication of recurrence, and all are alive and well (6).

## Conclusion

Asymptomatic cases of cholesterol granuloma are more difficult to diagnose than symptomatic cases, unless they are accidentally discovered during a preoperative CT or MRI chest screening. Most cases of cholesterol granuloma in the anterior mediastinum, or in the whole mediastinum, are asymptomatic, making it a rather uncommon disorder. Consequently cardiac or chest surgery often revealed all of those cholesterol granulomas. The most effective strategy for treatment is a complete surgical resection; after extirpation, there is no evidence that the condition will recur.

## Data Availability

The original contributions presented in the study are included in the article/Supplementary Material, further inquiries can be directed to the corresponding author.

## References

[1] Manabe T Oka S Ono K. Multifocal cholesterol granulomas of the anterior mediastinum. Surg Case Rep. (2020) 6(1):182. 10.1186/s40792-020-00943-5 32725385 PMC7387393

[2] Krishnan TR Sinha SK Kejriwal NK. A rare case of cholesterol granuloma in the anterior mediastinum. Heart Lung Circ. (2013) 22(4):303–4. 10.1016/j.hlc.2012.07.009 22906491

[3] Fujimoto K Takamori S Yano H Sadohara J Matsuo T Terazaki Y Focal cholesterol granuloma in the anterior mediastinum: [18F]-fluoro-2-deoxy-D-glucose-positron emission tomography and magnetic resonance imaging findings. J Thorac Oncol. (2007) 2(11):1054–6. 10.1097/JTO.0b013e318158eee8 17975500

[4] Drury NE Smith DN Phillips LM Trotter SE Kalkat MS. Cholesterol granuloma of the anterior mediastinum. Thorax. (2017 Jul) 72(7):671–2. 10.1136/thoraxjnl-2016-209481 27799631

[5] Hongo T Jiromaru R Kuga R Matsuo M Oda Y Nakagawa T. Cholesterol granuloma of the anterior mediastinum: a case report and literature review. Int J Surg Case Rep. (2023) 111:108852. 10.1016/j.ijscr.2023.108852 37734126 PMC10518478

[6] Weissferdt A Kalhor N Moran C. Primary thymic cholesteroloma: a clinicopathological correlation of four cases of an unusual benign lesion. Virchows Arch. (2015) 467(5):609–11. 10.1007/s00428-015-1822-8 26266777

